# DEVELOPMENT, RELIABILITY, AND VALIDITY OF THE CAREGIVER MEALTIME ENGAGEMENT SCALE IN NURSING HOMES

**DOI:** 10.1093/geroni/igz038.1741

**Published:** 2019-11-08

**Authors:** Wen Liu, Melissa Bachelar-Murphy, Kristine N Williams

**Affiliations:** 1 The University of Iowa, Iowa City, Iowa, United States; 2 George Washington University, Washington, D.C., United States; 3 University of Kansas Medical Center, Kansas City, Kansas, United States

## Abstract

Persons with dementia commonly experience mealtime challenging behaviors resulting in negative outcomes. Appropriate caregiver engagement is critical in engaging residents in eating. Current caregiver behavior measures are neither validated nor specific for mealtime care. A feasible and reliable measure to evaluate caregiver engagement during mealtimes is needed. Our team developed the Caregiver Mealtime Engagement Scale (CMES), a 29-item observational measure with good content validity (Content Validity Index = 1.00). The CMES includes 24 positive behaviors (e.g., position resident upright) and 5 negative behaviors (e.g., interrupt resident). Each item is scored by frequency on a 0 (never) – 3 (always) scale. Total score ranges from 0-87; higher score indicates better engagement. This study aimed to test the CMES’ reliability and validity through a secondary analysis of 87 mealtime video-recorded observations from a hand feeding trial (P30). The sample included 7 residents and 25 staff from 2 nursing homes. The CMES has good internal consistency (Cronbach’s α =.775). Inter-rater reliability was good (r = .861, p<.001) based on ratings of 20 videos by two independent trained coders. Intra-rater reliability was excellent (r = .905, p<.001) based on ratings of 20 videos by one trained coder at two times (2-3 weeks apart). The CMES demonstrated good convergent validity based on association with the Relational Behavior Scale (r = .822, p<.001) and Mealtime Relational Care Checklist (r = .324, p=.002). Findings support the CMES’ reliability and validity. Future research is needed to test CMES among a larger diverse sample of caregivers in different settings.

